# Up For A Challenge (U4C): Stimulating innovation in breast cancer genetic epidemiology

**DOI:** 10.1371/journal.pgen.1006945

**Published:** 2017-09-28

**Authors:** Leah E. Mechanic, Sara Lindström, Kenneth M. Daily, Solveig K. Sieberts, Christopher I. Amos, Huann-Sheng Chen, Nancy J. Cox, Marina Dathe, Eric J. Feuer, Michael J. Guertin, Joshua Hoffman, Yunxian Liu, Jason H. Moore, Chad L. Myers, Marylyn D. Ritchie, Joellen Schildkraut, Fredrick Schumacher, John S. Witte, Wen Wang, Scott M. Williams, U4C Challenge Participants, U4C Challenge Data Contributors, Elizabeth M. Gillanders

**Affiliations:** 1 Epidemiology and Genomics Research Program, Division of Cancer Control and Population Sciences, National Cancer Institute, National Institutes of Health, Bethesda, Maryland, United States of America; 2 Department of Epidemiology, University of Washington School of Public Health, Seattle, Washington, United States of America; 3 Sage Bionetworks, Seattle, Washington, United States of America; 4 Department of Biomedical Data Sciences, Dartmouth College, Lebanon, New Hampshire, United States of America; 5 Surveillance Research Program, Division of Cancer Control and Population Sciences, National Cancer Institute, National Institutes of Health, Bethesda, Maryland, United States of America; 6 Division of Genetic Medicine, Vanderbilt University, Nashville, Tennessee, United States of America; 7 Department of Biochemistry and Molecular Genetics, University of Virginia, Charlottesville, Virginia, United States of America; 8 Department of Epidemiology and Biostatistics, University of California San Francisco, San Francisco, California, United States of America; 9 Division of Informatics, Department of Biostatistics and Epidemiology, Institute for Biomedical Informatics, Perelman School of Medicine, University of Pennsylvania, Philadelphia, Pennsylvania, United States of America; 10 Department of Computer Science and Engineering, University of Minnesota-Twin Cities, Minneapolis, Minnesota, United States of America; 11 Department of Biochemistry and Molecular Biology, Center for Systems Genomics, Pennsylvania State University, University Park, Pennsylvania, United States of America; 12 Biomedical and Translational Informatics, Geisinger Health System, Danville, Pennsylvania, United States of America; 13 Department of Public Health Sciences, University of Virginia School of Medicine, Charlottesville, Virginia, United States of America; 14 Department of Population and Quantitative Health Sciences, Case Western Reserve University, Cleveland, Ohio, United States of America

**Fig 1:**
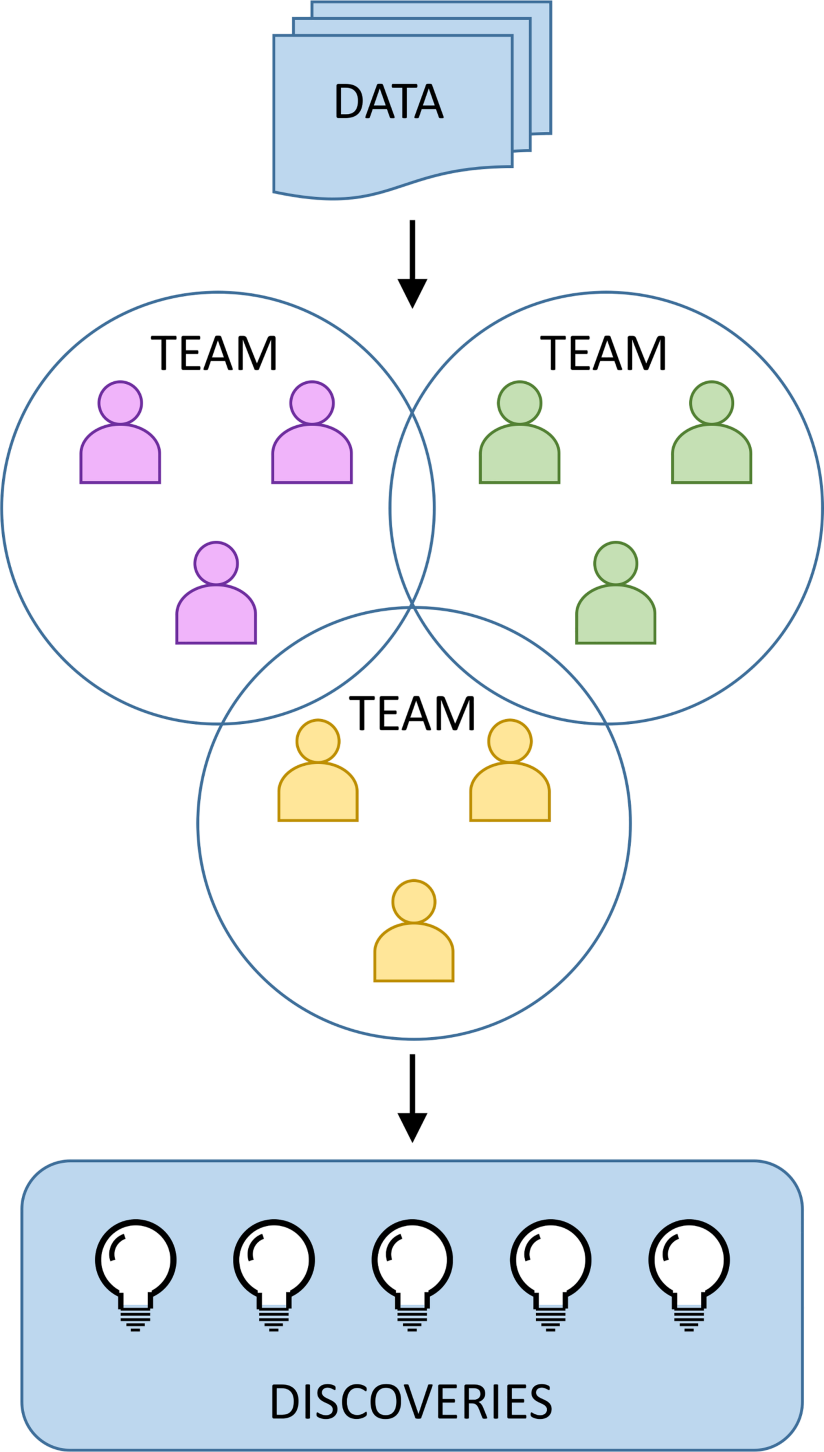
Stimulation of innovation in U4C. Existing genome-wide association studies (GWAS), representing thousands of cases and controls. Data were shared and accessed in a manner consistent with informed consent. Some of these data sets were made available for the first time in U4C. Teams competed for a prize to develop innovative analytical methods and make novel discoveries using these data sets.

Breast cancer remains a major public health burden, with an estimated 252,710 new cases and 40,610 deaths among women in the United States in 2017 [1]. To identify key genes and biological pathways potentially affecting disease risk, genome-wide association studies (GWAS) have been performed. At present, close to 100 common genetic variants have been associated with breast cancer [2–5]. However, these variants explain only a small proportion of the estimated genetic contribution to the risk of breast cancer [4]. GWAS analyses often report only results from single variant analyses, without exploring the impact of potential combinations or the interplay between variants. Therefore, in 2015, the National Cancer Institute (NCI) launched a challenge to inspire novel cross-disciplinary approaches to more fully decipher the genomic basis of breast cancer, called "Up For A Challenge (U4C)—Stimulating Innovation in Breast Cancer Genetic Epidemiology.” The goal of U4C was to promote the development and/or implementation of innovative approaches to identify novel risk pathways—including new genes or combinations of genes, genetic variants, or sets of genomic features—involved in breast cancer susceptibility in order to generate new biological hypotheses [6]. The challenge involved the formation of teams of scientists with diverse expertise to explore preexisting data sets, in an attempt to extract more useful information than typical GWAS analyses. U4C was also an explicit test of the usefulness of making larger data sets easily accessible to a broad community of researchers (Fig 1).

Fourteen teams, including 88 researchers, submitted 15 U4C entries. U4C participants applied several innovative approaches to the analysis of existing breast cancer GWAS data sets, leading to multiple novel findings (Table 1). After careful considerations from a scientific evaluation panel, the reproduction of primary findings based on in-house reanalyses by using the methods described in the entry, and a review by National Institutes of Health (NIH) judges, 3 entries were selected as U4C prize winners [6]. Team UCSF and UMN-CSBIO tied for the grand prize, Team Transcription was awarded second place, and U4C Maroons was the highest-scoring runner-up. Using their novel approaches, these teams discovered new genes by using a variety of analytical strategies, including imputing gene expression to perform gene-based association tests, network analyses, and the identification of variants that disrupt transcription factor (TF) binding associated with gene expression in breast tissue. The work of these 4 teams is now published as a series in *PLOS Genetics* to highlight the results of these truly innovative approaches to data reanalysis. Importantly, these papers passed the same rigorous editorial and external peer review evaluation that any submission to *PLOS Genetics* experiences.

**Table 1:** Overview of U4C entries.

Team Name	Entry Title	dbGaP Accession Number for U4C-Designated Data Sets Used	Other Data Sets	Strategy	Replication Strategy
Battalion Y	Integrative Analysis of Diverse Genomic Data Identifies Novel Link Between Immunity Pathways and Inherited Breast Cancer Risk	phs000147, phs000383, phs000517, phs000799, phs000812, phs000851, phs000912	GTEx, seeQTL, GenoSkyline, TCGA	GWAS, meta-analysis, functional annotation, protein–protein interaction, tissue specific enrichment, somatic mutations, eQTL analysis	Consistency across dbGaP data sets
CSMC_TEAM	Identifying Novel Genes and Pathways for Breast Cancer with Semiparametric Modeling	phs000147	Expression data from European Genome-Phenome Archive (EGAS00000000083), SNP gene annotation databases (KEGG, panther, cell map, BioCarta, etc.)	Linked SNPs to genes and pathways and looked for enrichment of genes and pathways in breast cancer	Compared dbGaP association results with gene expression and annotation databases
Gene Fishing	Novel Genetic Variants of Breast Cancer—SNPs, Genes, and Gene-Gene Interactions	phs000799	None	Linked SNPs to genes and performed gene-based and gene–gene interaction tests	Split data into testing and training
hapQTL	Haplotype Associations in Shanghai Breast Cancer Study (Up For A Challenge)	phs000799	None	Examined haplotype associations and identified nearby genes	Down sampling 100 times
MDACC	Association of X-Chromosome Genetic Variants and Breast Cancer Risk	phs000147, phs000383, phs000812, phs000851	snp-nexus.org; IPA Pathway Database	Single SNPs and gene-based tests of association on X chromosome and pathway analysis (IPA)	Consistency across studies and previous gene-expression publication
MDACC	Prediction of Breast Cancer Status Using X- Chromosome Genetic Variants	phs000147, phs000812, phs000851	snp-nexus.org; IPA Pathway Database	Single SNPS and gene-based random forests followed by pathway analysis (IPA)	Consistency across studies (used 1 study for training and others for testing)
muStat	Breast Cancer muGWAS	phs000147, phs000812	None	u-statistics for multivariate data (neighboring SNPs) integrating knowledge about genetics, leveraging information content and study-specific genome-wide significance	Consistency across studies (CGEMS, phs000147, and cohorts EPIC and PBCS of BPC3, phs000812) and consistency with published results from functional and expression data
snpsnbits	Identify Breast Cancer Pathways Using Iscore Screening	phs000147, phs000517, phs000799, phs000812, phs000851	NHGRI GWAS Catalogue, SNPedia	Used SNPs from literature and identified in GWAS to identify pathways (or gene sets) associated with breast cancer. Interactions and new SNPs were identified using an iscore	2 data sets for training, 3 data sets for testing
Team Transcription	Identification of Breast Cancer Associated Variants That Modulate Transcription Factor Binding	NHGRI GWAS Catalogue^a^	ENCODE, Roadmap Epigenomics, TCGA, GTEx	Integrative genomics approach included identifying transcription factor motifs and association with breast cancer, SNPs in LD with top GWAS, SNPs within motifs and DNase I hypersensitivity sites, and eQTL analysis	Consistency across multiple data sets and cell types
Team UCSF	Team UCSF Up For A Challenge Submission	phs000147, phs000383, phs000517, phs000799, phs000812, phs000851, phs000912	UK Biobank, GTEx	GWAS, GWAGE using PrediXscan, meta-analysis and admixture mapping	Replicated previous GWAS findings in the data sets, entry findings were replicated in UK biobank
U4C Maroons	U Chicago Maroons Project for U4C	phs000147, phs000383, phs000799, phs000812, phs000851	GAME-ON breast cancer GWAS summary statistics, Depression Genes and Network, and GTEx	GWAGE using MetxScan and meta-analysis	Consistency across data sets, replicated in GAME-ON breast cancer GWAS
UCLA Team	Multi-Ethnic Meta-Analysis and Fine Mapping in Breast Cancer	phs000383, phs000812, phs000851, phs000912	None	Mixed-model association, meta-analysis, forestPMplot, and fine mapping (CAVIAR)	Consistency across data sets
UMN-CSBIO	Genetic Interactions in Breast Cancer	phs000147, phs000812	Hapmap PhaseIII, Molecular Signatures Database (MSigDB v3.0) curated pathway database, Gene Annotation (hg19)	Pathway interactions using annotated gene sets from MSigDB v3.0	Replication in second data set (phs000147)
UNC-BIAS	U4C Breast Cancer Challenge	phs000147, phs000517, phs000799, phs000851	None	SNP and LD block-based (SNP set) association in subgroups and overall and meta-analysis	Consistency across data sets
UNH STATS	Data Mining of Genome-Wide Association Studies for New Hypotheses on the Possible Effect of Pathways on Breast Cancer Risk	phs000799, phs000851	None	Variable clustering, variable elimination and bootstrap forests	For 2 different studies, divided each study into training and validation sets, i.e., cross validation within study and replication among 2 studies

^a^This team used results from GWAS data as reported in the NHGRI GWAS catalogue (https://www.ebi.ac.uk/gwas/).

Abbreviations: BPC3, Breast and Prostate Cancer Cohort Consortium; CAVIAR, CAusal Variants Identification in Associated Regions; CGEMS, Cancer Genetic Markers of Susceptibility; dbGaP, Database of Genotypes and Phenotypes; ENCODE, Encyclopedia of DNA Elements; EPIC, European Prospective Investigation into Cancer; eQTL, expression quantitative trait loci; GAME-ON, Genetic Associations and Mechanisms in Oncology; GTEx, Genotype-Tissue Expression project; GWAGE, genome-wide association of gene expression; GWAS, genome-wide association studies; IPA, Ingenuity Pathway Analysis; iscore, influence score; LD, linkage disequilibrium; MSigDB, Molecular Signatures Database; NHGRI, National Human Genome Research Institute; PBCS, Polish Breast Cancer Case-Control Study; SNP, single nucleotide polymorphism; TCGA, The Cancer Genome Atlas; U4C, Up For A Challenge.

Team UCSF performed a genome-wide association of gene expression [7]. Using the gene-based association method PrediXcan [8], which integrates germline genotype and gene expression data, they identified novel associations between the following genes and breast cancer: *ACAP1*and *LRRC25* (using whole-blood transcriptome data) and *DHODH* (using breast- and mammary-tissue transcriptome data).

Team UMN-CSBIO applied a novel computational method, developed initially to analyze yeast data, called BridGE (Bridging Gene Sets with Epistasis) [9], to explicitly search for pathway-level interactions guided by annotated gene sets from the Molecular Signatures Database (MSigDB) [10]. By examining pathway interactions using 2 of the U4C-designated GWAS data sets, the team identified steroid hormone biosynthesis as a major hub of interactions and found that it was implicated as interacting with many pathways, including a gene set previously associated with acute myeloid leukemia (AML). These interactions would have been missed using traditional approaches.

Team Transcription employed an integrative genomics approach, exploring the hypothesis that many of the noncoding single nucleotide polymorphisms (SNPs) identified by GWAS alter TF binding sites and mediate the effect on disease by modulating TF binding and gene regulation [11]. This team identified a SNP, rs4802200, in perfect linkage disequilibrium (LD) with a GWAS-significant SNP (rs3760982). rs4082200 is predicted to disrupt ZNF143 binding within a breast cancer-relevant regulatory element. This SNP is a strong expression quantitative trait loci (eQTL) of *ZNF404* in breast tissue.

Team U4C Maroons also utilized a genome-wide gene expression approach, implemented in the MetaXcan [12], that leveraged GWAS summary statistics. This team identified *TP53INP2* (tumor protein p53-inducible nuclear protein 2), associated with estrogen-receptor–negative breast cancer. The association was consistent across 5 of the U4C GWAS data sets and in different populations (European, African, and Asian ancestry) [13].

U4C demonstrated that making breast cancer genetic epidemiologic data more widely available can accelerate breast cancer genetic epidemiologic research without necessarily generating more data. This was accomplished in a relatively brief period because the competition only ran for 8.5 months. Clearly, the success of the U4C necessitated the enhanced sharing of data and a concerted effort by many investigators from a wide variety of academic disciplines. The formation of new collaborations was encouraged as part of the challenge evaluation criteria, and the success of this multidisciplinary approach is evident in the uniqueness and strength of the results. Several U4C entries embraced the spirit of the competition by critically challenging genetic epidemiology norms. Such reexamination of existing paradigms within a field is important to intellectual growth, but given the inherent conservative nature of most disciplines, this is not always welcomed. We hope that activities such as U4C and the willingness of *PLOS Genetics* to evaluate and publish these types of studies will encourage more innovation that will generate more novel and important findings.

Another key reason for the success is that 7 breast cancer GWAS data sets were gathered and made available for the challenge via controlled access from the NIH data repository Database of Genotypes and Phenotypes (dbGaP) [14]. Such streamlined access to data promoted the success of U4C and is completely in agreement with the *PLOS Genetics* editorial policy [15]. In the future, an improved informed consent mechanism that explicitly enables analysis and reanalysis of data sets by multiple research teams could enhance the ability to pursue multidisciplinary approaches. This broad access also promoted the exploration of data across several continental ancestries. This is in contrast to the history of the genetic epidemiology of breast cancer, in which most GWAS have focused on populations of European descent, even though a few recent studies have highlighted the need to further explore initial findings in non-European populations [16–21]. With this in mind, U4C provided access to new non-European data sets to promote cross-ethnic analyses, and 9 U4C entries performed comparisons using populations of different ethnic groups, with several entries exploring approaches using non-European populations. Although the transethnic analyses were more complete than most studies in the past, not all groups leveraged all the available data, perhaps due in part to smaller numbers of understudied populations in available data sets. This will require improvement.

Overall, U4C successfully encouraged diverse research teams to expand analytical strategies in the genetic epidemiology of breast cancer and identify novel biological hypotheses for breast cancer risk. The approach leveraged a wide distribution of existing data sets that was a key and cost-effective means to furthering our understanding of breast cancer risk. Lastly, the results from U4C provide proof of principle that open competition can free investigators to push traditional boundaries and unleash their intellectual creativity to generate new and important insights into the biology of breast cancer and beyond.
